# 12-Month Post-Discharge Liver Function Test Abnormalities Among Patients With COVID-19: A Single-Center Prospective Cohort Study

**DOI:** 10.3389/fcimb.2022.864933

**Published:** 2022-04-14

**Authors:** Xuejiao Liao, Dapeng Li, Zhenghua Ma, Lina Zhang, Baoqi Zheng, Zhiyan Li, Guobao Li, Lei Liu, Zheng Zhang

**Affiliations:** ^1^ Institute for Hepatology, National Clinical Research Center for Infectious Disease, Shenzhen Third People’s Hospital, The Second Affiliated Hospital, School of Medicine, Southern University of Science and Technology, Shenzhen, China; ^2^ Shenzhen Research Center for Communicable Disease Diagnosis and Treatment of Chinese Academy of Medical Science, Shenzhen, China; ^3^ Guangdong Key Laboratory for Anti-Infection Drug Quality Evaluation, Shenzhen, China

**Keywords:** SARS-CoV-2, aspartate aminotransferase (AST), alanine aminotransferase (ALT), gamma-glutamyltransferase (GGT), albumin, COVID-19

## Abstract

**Objective:**

The longitudinal effects of severe acute respiratory syndrome coronavirus 2 (SARS-CoV-2) infection on the liver are unknown. This study aimed to characterize dynamic changes in liver function test abnormalities in patients with COVID-19 at the acute phase and recovery phase.

**Methods:**

A prospective cohort study involved patients with COVID-19 who were admitted to Shenzhen Third People’s Hospital between January 11, 2020, and April 27, 2020. Patients underwent liver function tests at hospitalization and at the outpatient visit at the 1-month, 3-month, 6-month, and 12-month follow-ups.

**Results:**

Among 461 patients, 28.4% of patients had any kind of liver function tests abnormality at admission, manifested as elevated ALT (13.0%), AST (17.6%), and GGT (15.8%) levels. The trajectory analysis indicated a marked improvement in liver function after discharge, with any kind of liver function test abnormalities of 25.1% at 1 month, 13.2% at 3 months, 16.7% at 6 months, and 13.2% at 12 months after discharge. Persistent liver function abnormalities were observed in patients with pre-existing conditions during follow-up. A significantly higher prevalence of ultrasound determined fatty liver disease was found in those patients with more frequent LFT abnormalities at follow-up.

**Conclusion:**

In this study of patients with COVID-19, liver damage in COVID-19 was usually temporary and could return to normal at the end of the 12-month follow-up.

## Introduction

Abnormal liver function is common in patients hospitalized with coronavirus disease 2019 (COVID-19), with a prevalence ranging from 15% to 65% (Marjot et al., 2021). A recent systematic review reported that the pooled prevalence of liver biochemistry abnormalities of any kind at admission was 46.9% (Del Zompo et al., 2020). During the clinical course of COVID-19, abnormal liver function at admission has been shown to be associated with the disease severity and mortality of SARS-CoV-2 infection, but the existing evidence is still controversial (Ding et al., 2021; Wang et al., 2021; Weber et al., 2021). By the time of discharge, a study reported that 40.50% of patients still had abnormal liver function tests (Gan et al., 2021). The long-term symptoms and disability of convalescent patients with COVID-19 are common and have been termed long COVID-19 (Nalbandian et al., 2021; Shah et al., 2021). Cumulative clinical evidence suggests that severe acute respiratory syndrome coronavirus 2 (SARS-CoV-2) infection may be followed by long-term abnormalities in multiple organ systems, including the liver (Gupta et al., 2020). A recent study, including patients recovering from COVID-19 in Wuhan, showed that 7.6% of patients still had abnormal liver function at the end of 12 months follow-up (Liu et al., 2021). However, few studies have comprehensively assessed hepatobiliary sequelae and dynamic changes in liver function among COVID-19 survivors after hospital discharge. Therefore, this prospective cohort study aimed to evaluate the temporal changes in liver function among COVID-19 patients during hospitalization and over 12 months of follow-up.

## Methods

### Population and Design

This study was approved by the Ethics Committee of Shenzhen Third People’s Hospital (IRB 2020-021-02). Written informed consent was obtained from all patients. This is a prospective cohort study conducted among patients recruited by Shenzhen Third People’s Hospital. Our hospital is a tertiary referral hospital in southern China that is responsible for providing medical services for infectious diseases. The hospital has a total of 2,608 beds, including 1,008 negative pressure isolation rooms. After the outbreak of SARS-CoV-2, our hospital was designated as the only hospital for COVID-19 treatment in Shenzhen city. According to the current policy of disease prevention and control for COVID-19, all patient including non-severe COVID-19 will be admitted to our hospital after they are tested positive. Patients with COVID-19 were diagnosed with quantitative reverse transcriptase-polymerase chain reaction (qRT-PCR) by the key laboratory of Shenzhen Centers for Disease Control and Prevention (CDC). This study included patients who were hospitalized between January 11 and April 27, 2020.

Demographic information (age, sex), smoking history, comorbidities (including diabetes, hypertension, cardiovascular disease, and hepatitis B infection), laboratory data, and clinical outcomes [intensive care unit (ICU) admission, death, and length of hospital stay] were collected from the electronic medical record system. According to the National Health Commission of China guidance for the management of COVID-19, disease severity was divided into four severity grades (mild, moderate, severe, or critical). Patients were discharged after meeting uniform standards: 1) body temperature is back to normal for more than three days; 2) respiratory symptoms improve obviously; 3) pulmonary imaging shows obvious absorption of inflammation; 4) nuclei acid tests negative twice consecutively on respiratory tract samples. Participants were followed up at 1 month, 3 months, 6 months, and 12 months after hospital discharge between February 21, 2020 and July 10, 2021. During each visit, liver enzymes were evaluated, including aspartate aminotransferase#146; (AST), alanine aminotransferase (ALT), gamma-glutamyltransferase (GGT), and albumin. Any kind of liver function test (LFT) abnormality was defined as the elevation of ALT, AST, or GGT that was higher than the upper limit of normal (ULN). The ULN was as follows: ALT>40 U/L, AST>40 U/L, GGT>49 U/L. Hypoalbuminemia was defined as a blood level of albumin < 35 g/L. At each outpatient visit, hepatoprotective drugs were prescribed for patients with ALT > 2 ULN. In addition, participants received liver ultrasound to assess diffuse liver disease, including fatty liver, at the end of follow-up.

### Statistical Analysis

Categorical variables are expressed as numbers (n) and percentages (%). Continuous variables are represented by the median and interquartile range (IQR). Student’s t-test or Mann–Whitney U test for continuous variables and Chi-square test or Fisher’s exact test for categorical variables were performed to determine the group differences. All statistical analyses were performed with R version 4.1.0. Statistical significance was set as a two-sided *P* value less than 0.05.

## Result

### Population Characteristics

**Figure 1:**
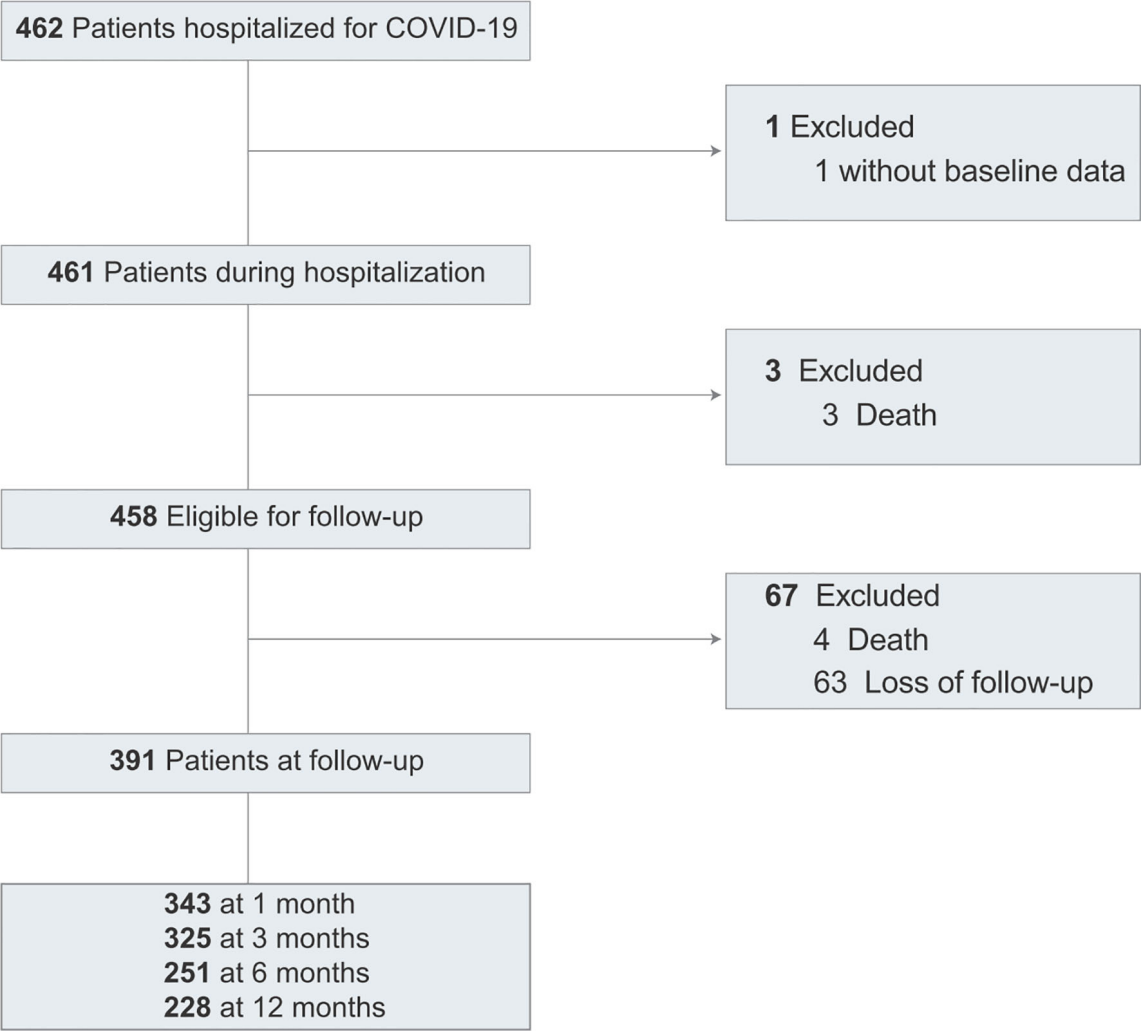
Study flow diagram.

Of 462 patients, 461 patients were included in the study (**Figure 1**). The baseline clinical characteristics of COVID-19 patients are presented in **Table 1**. The median age was 45 years (IQR, 32-59); 230 (49.9%) were women; 92 (20.0%) had severe or critical illness. Thirty-one (6.7%) patients were admitted to the ICU, and the median length of the ICU stay was 18 days. In the hospitalization, 3 patients died. The median length of hospital stay was 21 days for 458 discharged patients.

**Table 1:** Baseline Characteristics in Included Patients with COVID-19.

Characteristic	N = 461
Age, median (IQR), y	45 (32 – 59)
Sex, female, n (%)	230 (49.9)
Body mass index, median (IQR), kg/m²	23.3 (21.2 – 25.5)
Comorbidities, n (%)	100 (21.7)
Hypertension, n (%)	55 (11.9)
Hepatitis B infection, n (%)	35 (7.6)
Diabetes, n (%)	21 (4.6)
Cardiovascular disease, n (%)	13 (2.8)
Cancer, n (%)	5 (1.1)
Disease severity, severe/critical, n (%)	92 (20.0)
ICU admission, n (%)	31 (6.7)
Duration of ICU stay, median (IQR), d	18 (10 – 28)
Death, n (%)	3 (0.7)
Hospital stays, median (IQR), d	21 (15 – 28)

IQR, interquartile range; ICU, intensive care unit.

### Liver Function Test Abnormalities During Hospitalization

Of 461 patients, 122 patients (28.4%) had any kind of LFT abnormalities at admission (including ALT, AST, and GGT). The elevations of ALT, AST, and GGT at admission were 13.0%, 17.6%, and 15.8%, respectively. Hypoalbuminemia was found in 1.5% of patients (**Table S1**). The changes in LFT abnormalities, ALT, AST, GGT, and albumin during hospitalization are presented in **Figure S1**. LFT abnormalities were common in COVID-19 during hospitalization. A significant increase in the proportion of any kind of LFT abnormality, mainly GGT elevation and ALT elevation, was identified during hospitalization, but a reduction in AST. The dynamic change in liver function was characterized by a dual pattern after admission, which was the elevation of ALT and AST followed by the elevation of ALT and GGT.

By the time of discharge, 34.5% (158 cases) had LFT abnormalities among 458 discharged patients (**Table S1**). Detailed liver chemistry results showed that GGT elevation (114 cases, 24.9%) and ALT elevation (95 cases, 20.7%) were most common. Hypoalbuminemia was observed in 2.0% of patients. The rate of ALT elevation and GGT elevation increased significantly compared to those at admission, and the rate of AST elevation decreased significantly.

### Liver Function Test Abnormalities During Follow-Up

Of the 458 discharged patients, 4 died within 12 months after discharge. A total of 391 patients underwent at least one liver function test at the 1-month, 3-month, 6-month, and 12-month follow-ups. Liver function tests were performed in 1,147 blood samples from 391 COVID-19 patients during the follow-up period. The median duration from discharge to outpatient visit was 30 days (IQR, 28-33) for 1 month, 90 days (IQR, 76-94) for 3 months, 184 days (IQR, 180-189) for 6 months, and 377 days (IQR, 355-398) for 12 months. At each outpatient visit, hepatoprotective drugs of compound glycyrrhizin tablets or glutathione were prescribed for patients with LFT abnormalities. Liver biopsy were performed in 4 patients with Hepatitis B infection for evaluating hepatic pathology.

Most patients showed an improvement in their liver function tests after discharge (**Table 2**). At 1 month, 25.1% (86 of 343) of patients had any kind of LFT abnormality, with ALT elevation (14.9%, 51 of 343) and GGT elevation (14.6%, 50 of 343) being the most common. At 3 months, 13.2% (43 of 325) of patients had any kind of LFT abnormality, with 8.3% (27 of 325) of ALT elevation and 8.0% (26 of 325) of GGT elevation. At 6 months, 16.7% (42 of 251) of patients had any kind of LFT abnormality, with ALT elevation (11.2%, 28 of 251) and GGT elevation (9.6%, 24 of 251). At 12 months, the proportion of any kind of LFT abnormality decreased to 13.2% (30 of 228), with a predominant elevation of GGT (8.3%, 19 of 228). In addition, these liver function tests were minimally elevated (1-2×ULN) at follow-up. Hypoalbuminemia was not observed at any time point of follow-up.

**Table 2:** Liver Function Tests Abnormalities in Patients with COVID-19 at the Time of Follow-up.

Variable	1 month	3 months	6 months	12 months	*P* value
n	343	325	251	228	
Any kind, n (%)	86 (25.1%)	43 (13.2%)	42 (16.7%)	30 (13.2%)	<0.001
AST, median (IQR), U/L	22 (18, 27)	21 (17, 25)	21 (17, 25)	19 (16, 23)	<0.001
AST elevation, n (%)					
Normal	330 (96.2%)	314 (96.6%)	241 (96.0%)	224 (98.2%)	
1-2 × ULN	12 (3.5%)	11 (3.4%)	9 (3.6%)	3 (1.3%)	
2-5 × ULN	1 (0.3%)	0 (0.0%)	1 (0.4%)	1 (0.4%)	
> 5 × ULN	0 (0.0%)	0 (0.0%)	0 (0.0%)	0 (0.0%)	
ALT, median (IQR), U/L	20 (13, 32)	18 (12, 25)	18 (13, 29)	17 (12, 23)	0.006
ALT elevation, n (%)					
Normal	292 (85.1%)	298 (91.7%)	223 (88.8%)	212 (93.0%)	
1-2 × ULN	44 (12.8%)	22 (6.8%)	22 (8.8%)	14 (6.1%)	
2-5 × ULN	7 (2.0%)	5 (1.5%)	6 (2.4%)	2 (0.9%)	
> 5 × ULN	0 (0.0%)	0 (0.0%)	0 (0.0%)	0 (0.0%)	
GGT, median (IQR), U/L	27 (18, 40)	22 (15, 31)	21 (14, 33)	20 (13, 29)	<0.001
GGT elevation, n (%)					
Normal	293 (85.4%)	299 (92.0%)	227 (90.4%)	209 (91.7%)	
1-2 × ULN	41 (12.0%)	24 (7.4%)	18 (7.2%)	17 (7.5%)	
2-5 × ULN	8 (2.3%)	1 (0.3%)	5 (2.0%)	2 (0.9%)	
> 5 × ULN	1 (0.3%)	1 (0.3%)	1 (0.4%)	0 (0.0%)	
Albumin, median (IQR), g/L	46.50 (44.90, 48.10)	46.40 (45.00, 47.70)	46.70 (45.20, 48.00)	47.45 (45.77, 48.90)	<0.001
Hypoalbuminemia, n (%)	0 (0.0%)	0 (0.0%)	0 (0.0%)	0 (0.0%)	

IQR, interquartile range; ULN, upper limit of normal; ALT, alanine aminotransferase; AST, alkaline phosphatase; GGT, gamma-glutamyltransferase.

### Persistent Liver Function Abnormalities After Discharge

**Figure 2:**
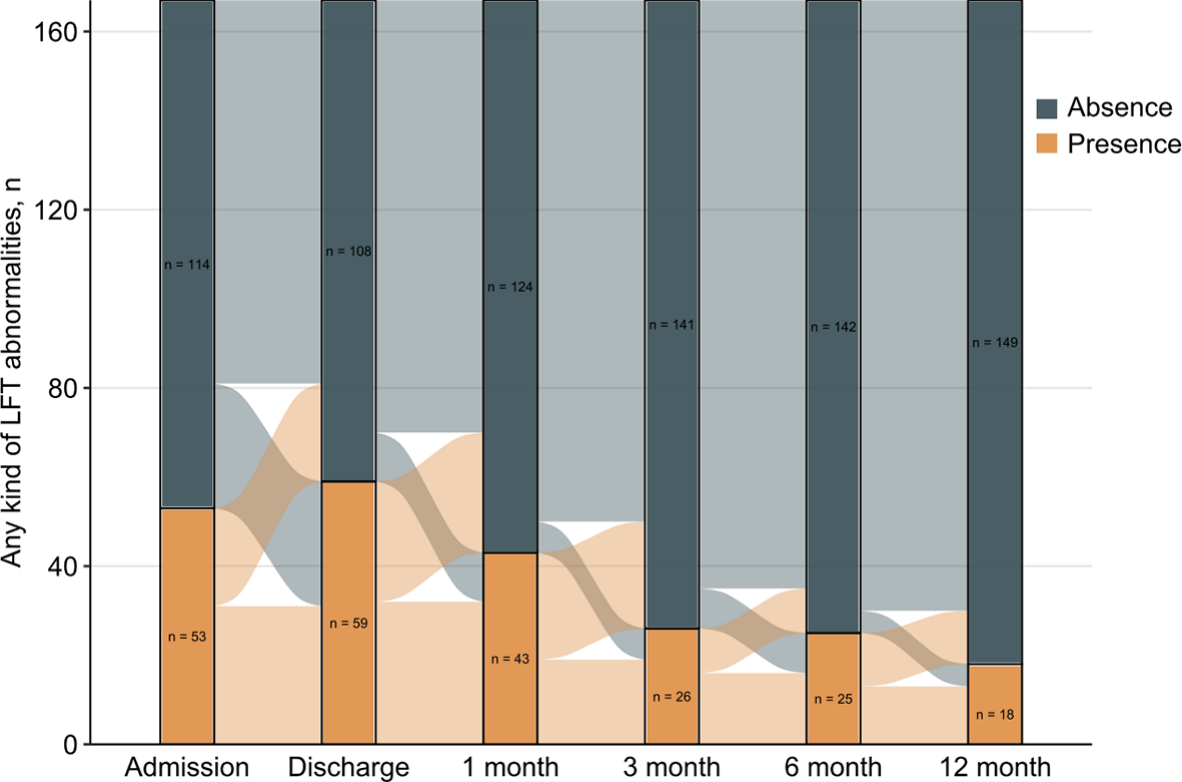
Evolution of liver function tests abnormalities over time for 167 patients with complete data in the study period.

A total of 167 patients had complete data on liver function tests at admission and discharge as well as all 4 follow-up time points. **Figure 2** describes the evolution of any kind of LFT abnormality. Most patients showed a significant improvement in their liver function at each time point of 1 month, 3 months, 6 months, and 12 months. Among the 167 participants, the proportions of LFT abnormalities at discharge and at the 1-month, 3-month, 6-month and 12-month follow-ups were 35.3%, 25.7%, 15.6%, 15.0%, and 10.8%, respectively. On the other hand, 107 (64.1%) participants had no LFT abnormalities at all 4 follow-up time points, and 44 (26.3%) had one or two abnormalities. Finally, 16 (9.6%) participants had three or four abnormalities. Comparisons in patients with normal and LFT abnormalities after discharge were performed (**Table 3**). Persistent abnormalities were more common in males with higher BMI and baseline LFT abnormalities during follow-up. We also found a significant difference in ultrasound findings of the liver at the 12-month follow-up. There was a significantly higher prevalence of ultrasound determined fatty liver disease in those patients with more frequent LFT abnormalities at follow-up.

**Table 3:** Comparison of Demographics, Comorbidities, Outcomes, Baseline Liver Biochemistry, and Ultrasound Results between Subgroups Stratified by the Number of Liver Function Tests Abnormalities at 4 Follow-up Time Points.

Variable	0 times	1-2 times	3-4 times	*P* value
n	107	44	16	
Age, median (IQR), y	45 (35, 57)	44 (35, 51)	42 (36, 55)	0.900
Sex, male, n (%)	41 (38.3%)	26 (59.1%)	14 (87.5%)	<0.001
Body mass index, median (IQR), kg/m² ^†^	22.9 (21.0, 25.1)	23.9 (21.5, 26.0)	25.8 (23.5, 27.1)	0.003
Any kind of comorbidities, n (%)	24 (22.4%)	15 (34.1%)	1 (6.2%)	0.072
Hepatitis B infection	8 (7.5%)	7 (15.9%)	0 (0.0%)	0.120
Disease severity, severe/critical, n (%)	18 (16.8%)	5 (11.4%)	7 (43.8%)	0.021
LFT abnormality at admission, n (%)	20 (18.7%)	18 (40.9%)	15 (93.8%)	<0.001
LFT abnormality at discharge, n (%)	21 (19.6%)	24 (54.5%)	14 (87.5%)	<0.001
Ultrasound findings of liver at 12 months ^‡^				
Parenchymal echo				<0.001
Normal	74 (80.4%)	32 (74.4%)	4 (28.6%)	
Fatty liver	17 (18.5%)	9 (20.9%)	10 (71.4%)	
Coarsened echo	1 (1.1%)	2 (4.7%)	0 (0.0%)	
Oblique diameter of right lobe	129 (116, 137)	135 (128, 141)	142 (130, 152)	0.003

^†^ 7 missing, ^‡^ 18 missing.

IQR, interquartile range; ICU, intensive care unit; LFT, liver function tests.

## Discussion

This is a comprehensive study on longitudinal monitoring of liver function in patients with COVID-19 for up to 12 months post-hospital discharge. Liver damage in COVID-19 was usually temporary and could return to normal at the end of the 12-month follow-up. Patients with pre-existing conditions were more likely to have persistent liver function abnormalities during follow-up.

Studies have reported that LFT abnormalities are common in patients with COVID-19, with a wide range of rates. A meta-analysis including Chinese studies reported that the pooled rate of both AST and ALT elevation was 15.0% (Sultan et al., 2020). In this study, AST and ALT elevations at admission were observed in 13.0% and 17.6% of patients, respectively. However, studies from the United States have reported a higher rate of LFT abnormalities (Cholankeril et al., 2020; Hajifathalian et al., 2020; Hundt et al., 2020; Team, 2020). A study in Germany revealed that ALT and AST elevation at the time of hospital admission was observed in 27% and 42%, respectively (Weber et al., 2021). The heterogeneity in the rate of LFT abnormalities might be attributed to the difference in the time from infection to hospital admission. In China, most patients are hospitalized at the early stages of infection through large-scale screening of close contacts.

Previous studies have shown that abnormal liver function tests are mainly caused by AST elevation and ALT elevation (Lei et al., 2020; Bloom et al., 2021; Herta and Berg, 2021). Compared with AST and ALT, GGT elevation is less common. Contrary to these studies, our study suggested that GGT elevation was one of the most important LFT abnormalities throughout the hospital stay. A dual pattern of abnormal liver function can be observed during hospitalization, which was characterized by an increase in ALT and AST in the early stage, followed by an increase in ALT and GGT in the later stage. GGT is a diagnostic biomarker of cholangiocyte injury, which has been less reported in previous studies (Shao et al., 2020). Our study revealed that GGT elevation has become a major indicator of abnormal liver function at the time of discharge. The pathogenic mechanism of liver involvement caused by SARS-CoV-2 may be multifactorial, including viral infection of the liver, systemic inflammation caused by cytokine storms, drug-induced liver injury, and hypoxemia associated with pneumonia (Yu et al., 2021). ACE2 has been determined to be the receptor for SARS-CoV-2 to infect host cells, which were highly expressed in bile duct cells of healthy individuals by our analysis of single-cell RNA sequencing data (Qi et al., 2020). Another study also found low expression of ACE2 in hepatocytes (2.6%), and the average expression level was 20 times lower than that in bile duct cells (Chai et al., 2020). Therefore, SARS-CoV-2 may induce liver damage *via* the effect of cholangiocytes.

Recent views have raised concerns about long-term multiorgan sequelae, including liver sequelae (Nalbandian et al., 2021). Given the huge need for health care for patients with sequelae of COVID-19, recognition of the different organ sequelae of SARS-CoV-2 is very important for follow-up management and medical resource allocation. Few studies have reported liver function in recovered patients with COVID-19. A recent study, which defined abnormal liver function with ALT or AST values above the ULN, reported that 7.6% of patients still showed abnormal liver function at 12 months after discharge (Liu et al., 2021). Our study showed consistent results: 5.4% of recovered patients with COVID-19 had ALT or AST elevation at the end of the 12-month follow-up. We additionally showed that GGT elevation was the main LFT abnormality in the long-term follow-up. Moreover, LFT abnormalities usually presented with minimal elevation (1-2×ULN) in our follow-up cohort, and liver damage in most recovery cases is usually mild and temporary and can return to normal within a short time during the recovery period. These results suggested that COVID-19 disease or the existing drugs may not cause long-term serious liver damage for most patients. Our study suggested that a subgroup of patients had persistent LFT abnormalities during the recovery period, although hepatoprotective drugs were given after the presence of abnormalities. This study observed that a higher prevalence of fatty liver disease in recovery patients with more frequent LFT abnormalities. And persistent LFT abnormalities were more common in males with higher BMI and baseline LFT abnormalities. Previous studies showed that approximately 10% to 20% of otherwise healthy patients may have abnormal LFTs (Radcke et al., 2015; Wang et al., 2017). Although fatty liver disease may occur after COVID-19 disease in some patients, it cannot be excluded that these recovery patients had abnormal LFTs at the acute phase and recovery phase due to the interaction of pre-existing fatty liver disease and COVID-19. Moreover, chronic liver disease, including chronic viral hepatitis, nonalcoholic fatty liver disease, and alcohol-related liver disease, is a major disease burden in China, which affected approximately 300 million people (Wang et al., 2014). Given the high burden, the long-term effects of COVID-19 on underlying chronic liver disease require detailed assessment.

This study has several limitations. First, the single-center design may introduce selection bias. According to the policy for the treatment of COVID-19, all confirmed patients in Shenzhen city were admitted to our hospital. Patients with less than moderate COVID-19 disease do not generally get admitted to the hospital in western countries. Compared with previous studies including patients with severe illness, this study analyzed the changes in liver functions of patients with different disease severities. Second, more patients were lost to follow-up over time. By dynamic analysis in 167 patients with complete data at different time points, this problem was alleviated. Third, the association between LFT abnormalities and mortality cannot be assessed, as 4 recovery patients died in this follow-up cohort. Future research should continue to study the potential impact of SARS-CoV-2 on long-term mortality in patients recovering from COVID-19.

## Conclusion

Our study suggested that the patient’s liver function significantly improved from discharge to the 12-month follow-up. A subset of recovered patients with pre-existing conditions presented persistent LFT abnormalities. Clinical significance of the mild persistent LFT abnormalities should be interpreted with caution.

## Data Availability Statement

The original contributions presented in the study are included in the article/**Supplementary Material**. Further inquiries can be directed to the corresponding authors.

## Ethics Statement

The studies involving human participants were reviewed and approved by Ethics Committee of Shenzhen Third People’s Hospital. Written informed consent to participate in this study was provided by the participants’ legal guardian/next of kin.

## Author Contributions

ZZ, LL, and GL conceptualized and designed the study, and reviewed and revised the manuscript. XL and DL designed the data collection instruments, and performed data analyses, interpreted data, and drafted the initial manuscript. ZM, LZ, BZ, and ZL collected data, performed clinical examination, and carried out the initial analyses, and reviewed and revised the manuscript. All authors approved the final manuscript as submitted and agreed to be accountable for all aspects of the work.

## Funding

This study was supported by the National Science Fund for Distinguished Young Scholars (82025022), the Science and Technology Innovation Committee of Shenzhen Municipality (JSGG20200207155251653), the Emergency Key Program of Guangzhou Laboratory (EKPG21-29), and the Central Charity Fund of Chinese Academy of Medical Science (2020-PT310-009).

## Conflict of Interest

The authors declare that the research was conducted in the absence of any commercial or financial relationships that could be construed as a potential conflict of interest.

## Publisher’s Note

All claims expressed in this article are solely those of the authors and do not necessarily represent those of their affiliated organizations, or those of the publisher, the editors and the reviewers. Any product that may be evaluated in this article, or claim that may be made by its manufacturer, is not guaranteed or endorsed by the publisher.
