# Supplementary material for: 12-Month Post-Discharge Liver Function Test Abnormalities Among Patients With COVID-19: A Single-Center Prospective Cohort Study
Source: Front Cell Infect Microbiol. 2022 Apr 14;12:864933. doi: 10.3389/fcimb.2022.864933 (PMC9046934; doi:10.3389/fcimb.2022.864933)
Supplement: Supplementary file 1 [file DataSheet_1.docx]

Supplementary Material

# Supplementary Tables and Figures

## Supplementary Tables

**Table S1. Comparison of liver function tests between admission and discharge.**

| **Variable** | **Admission** | **Discharge** | ***P* value** |
| --- | --- | --- | --- |
| n | 461 | 458 |  |
| Any kind, n (%) | 131 (28.4%) | 158 (34.5%) | 0.047 |
| AST, median (IQR), U/L | 26 (21, 35) | 21 (17, 28) | <0.001 |
| AST elevation, n (%) | 81 (17.6%) | 33 (7.2%) | <0.001 |
| ALT, median (IQR), U/L | 21 (15, 31) | 22 (13, 36) | 0.200 |
| ALT elevation, n (%) | 60 (13.0%) | 95 (20.7%) | 0.002 |
| GGT, median (IQR), U/L | 23 (16, 36) | 28 (19, 49) | <0.001 |
| GGT elevation, n (%) | 73 (15.8%) | 114 (24.9%) | <0.001 |
| Albumin, median (IQR), g/L | 43.4 (41.3, 45.8) | 41.7 (39.2, 43.8) | <0.001 |
| Hypoalbuminemia, n (%) | 7 (1.5%) | 9 (2.0%) | 0.600 |

IQR: interquartile range; ALT: alanine aminotransferase; AST: alkaline phosphatase; GGT: gamma-glutamyltransferase

## Supplementary Figures





**Figure S1. Evolution of liver function tests abnormalities during hospitalization.**

ALT: alanine aminotransferase; AST: alkaline phosphatase; GGT: gamma-glutamyltransferase
